# Supplementary material for: Polo-like kinase 1 prevents excess microtubule polymerization in C. elegans oocytes to ensure faithful meiosis
Source: bioRxiv. 2024 Aug 4:2024.08.03.606476. Preprint. [Version 1] doi: 10.1101/2024.08.03.606476 (PMC11312516; doi:10.1101/2024.08.03.606476)
Supplement: Supplement 1 [file NIHPP2024.08.03.606476v1-supplement-1.pdf]

bioRxiv preprint doi: https://doi.org/10.1101/2024.08.03.606476; this version posted August 4, 2024. The copyright holder for this preprint (which was not certified by peer review) is the author/funder, who has granted bioRxiv a license to display the preprint in perpetuity. It is made available under aCC-BY 4.0 International license.

FIGURE S1

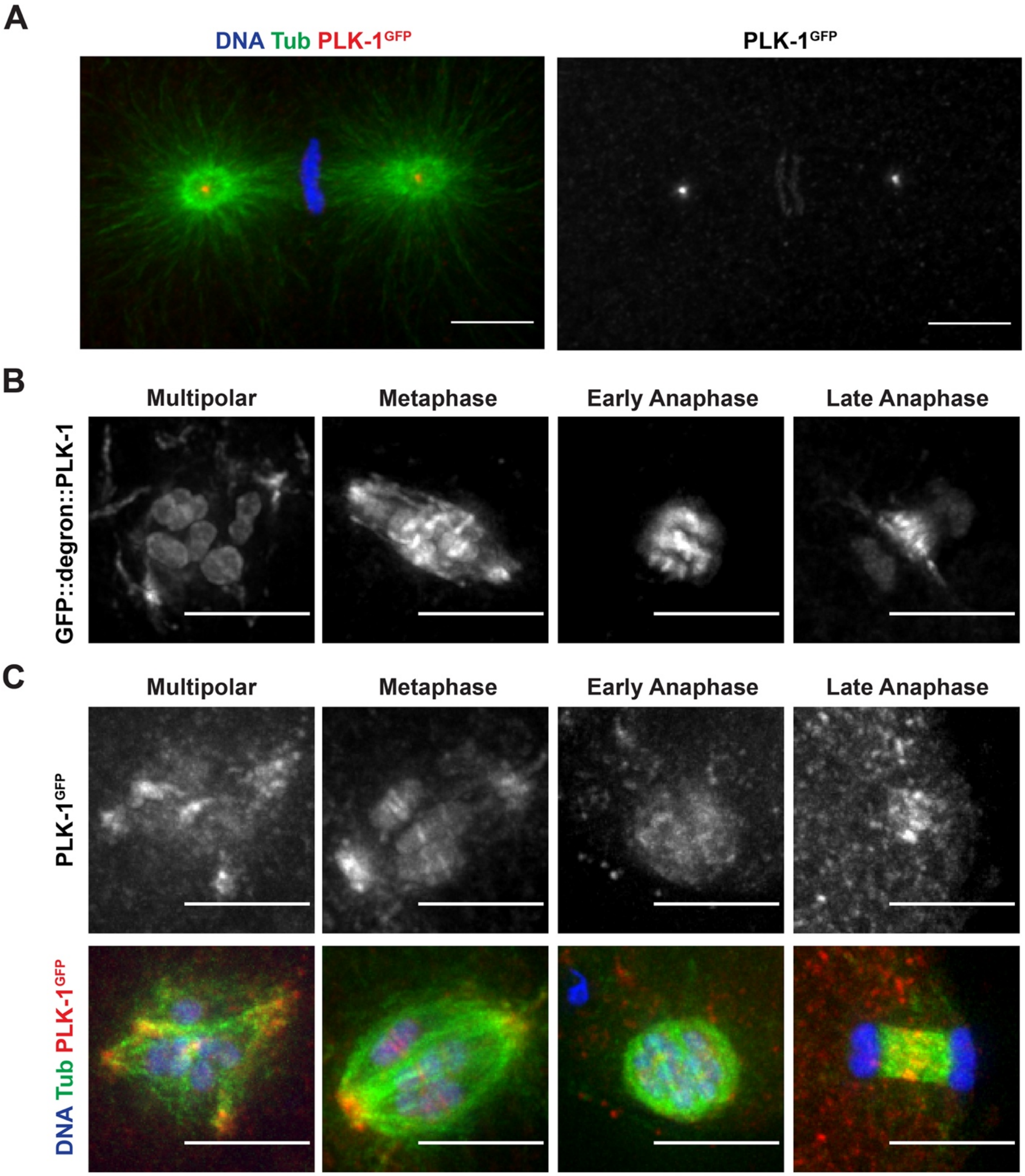

bioRxiv preprint doi: https://doi.org/10.1101/2024.08.03.606476; this version posted August 4, 2024. The copyright holder for this preprint (which was not certified by peer review) is the author/funder, who has granted bioRxiv a license to display the preprint in perpetuity. It is made available under aCC-BY 4.0 International license.

Figure S1: Localization of PLK-1 on the meiotic spindle during various stages of oocyte meiosis using an AID strain is consistent with existing literature

A) Immunofluorescence of an embryo from the PLK-1 AID strain using a GFP antibody shows that PLK-1 localizes to the centrosomes and kinetochores during mitosis. Shown are DNA (blue), tubulin (green) and PLK-1 (red).

B) Endogenous localization of GFP in ethanol fixed whole worms expressing GFP::degron::PLK-1 shows that PLK-1 localizes to the spindle poles, ring complex, DNA, kinetochore cups and kinetochore filaments during the multipolar and metaphase stages. During early anaphase, PLK-1 is present on the DNA and ring complex, and during late anaphase PLK-1 is on the spindle midzone and diffusely on the DNA.

C) Immunofluorescence on untreated, dissected oocytes shows that PLK-1 localizes to the spindle poles, ring complex, DNA and spindle midzone during various stages of oocyte meiosis. Shown are DNA (blue), tubulin (green) and PLK-1 (red, stained with a GFP antibody). All scale bars = 5 μm.

bioRxiv preprint doi: https://doi.org/10.1101/2024.08.03.606476; this version posted August 4, 2024. The copyright holder for this preprint (which was not certified by peer review) is the author/funder, who has granted bioRxiv a license to display the preprint in perpetuity. It is made available under aCC-BY 4.0 International license.

FIGURE S2

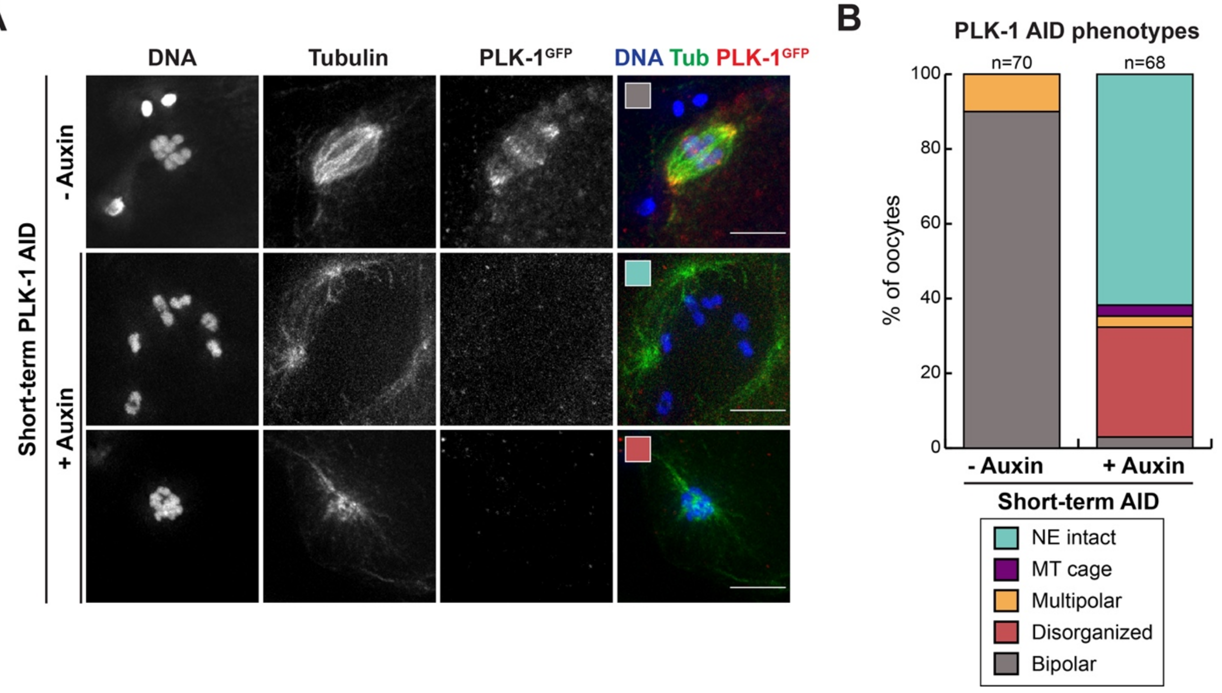

Figure S2: Short-term PLK-1 depleted oocytes fail to assemble and maintain bipolar spindles

A) Control and short-term auxin treated PLK-1 AID unarrested oocytes were stained for DNA (blue), tubulin (green) and PLK-1 (using a GFP antibody; red). Representative images of the major phenotypic categories quantified in B are indicated with colored boxes. Scale bars = 5 µm.

B) Quantification of the experiment shown in A. Control spindles were largely bipolar, while short-term auxin treated oocytes were either disorganized or had an intact nuclear envelope, consistent with long-term AID.

bioRxiv preprint doi: https://doi.org/10.1101/2024.08.03.606476; this version posted August 4, 2024. The copyright holder for this preprint (which was not certified by peer review) is the author/funder, who has granted bioRxiv a license to display the preprint in perpetuity. It is made available under aCC-BY 4.0 International license.

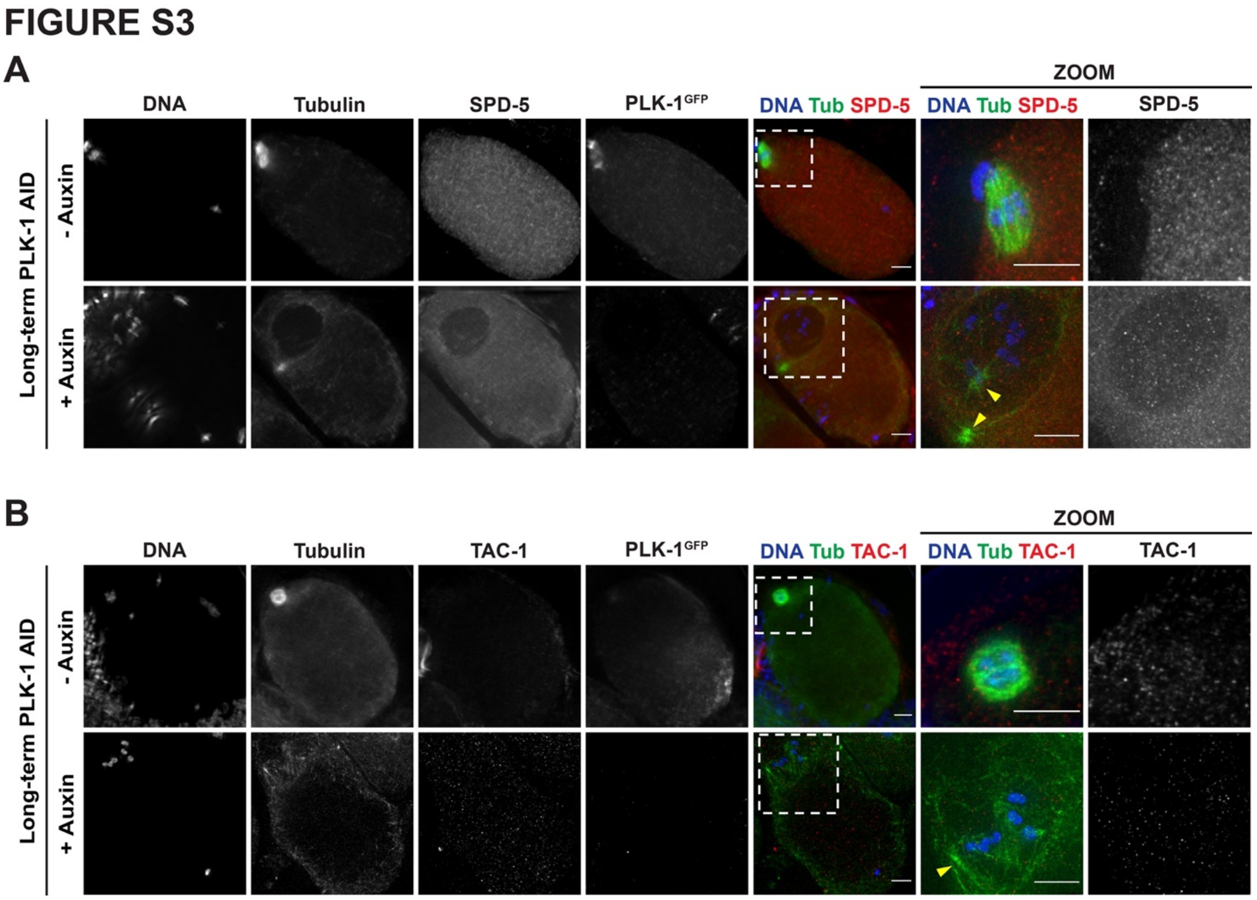

Figure S3: PCM components SPD-5 and TAC-1 are not present on oocyte chromosome-adjacent asters

Control and long-term auxin treated PLK-1 AID oocytes were stained for DNA (blue), tubulin (green), SPD-5 (red) and PLK-1 (using a GFP antibody; not shown in merge) (A) or DNA (blue), tubulin (green), TAC-1 (red) and PLK-1 (using a GFP antibody; not shown in merge) (B) and imaged at 40X (columns 1-5) and 100X magnification (zooms). SPD-5 localized to 0/33 asters (arrowheads) observed near the oocyte chromosomes in long-term auxin treated oocytes, while TAC-1 localized to 1/16 asters. Scale bars = 5 µm.

bioRxiv preprint doi: https://doi.org/10.1101/2024.08.03.606476; this version posted August 4, 2024. The copyright holder for this preprint (which was not certified by peer review) is the author/funder, who has granted bioRxiv a license to display the preprint in perpetuity. It is made available under aCC-BY 4.0 International license.

SUPPLEMENTAL VIDEO LEGENDS

Video 1: Metaphase-arrested oocyte spindles in the PLK-1 AID strain maintain bipolarity in the absence of auxin

Live imaging of an *emb-30(RNAi)* metaphase-arrested oocyte spindle; corresponds to Figure 2C (top row). Shown are GFP::tubulin and GFP::PLK-1 (green), and mCherry::histone (magenta). Oocytes were dissected into Meiosis Media containing vehicle and immediately filmed. The spindle maintains bipolarity; chromosomes oscillate between either pole and the midspindle retains its integrity. This phenotype was consistent in all videos (n=7). Scale bar = 5μm.

Video 2: Metaphase-arrested oocyte spindles in the PLK-1 AID strain elongate and lose midspindle integrity upon acute auxin treatment

Live imaging of an *emb-30(RNAi)* metaphase-arrested oocyte spindle treated with auxin; corresponds to Figure 2C (bottom row). Shown are GFP::tubulin and GFP::PLK-1 (green), and mCherry::histone (magenta). Oocytes were dissected into auxin-containing Meiosis Media and immediately filmed. The spindle poles immediately move apart, the chromosomes lose alignment, and the midspindle splays resulting in a disorganized spindle. This phenotype was consistent in all videos (n=6). Scale bar = 5μm.

Video 3: Unarrested oocyte spindles maintain bipolarity and undergo anaphase in the absence of auxin

Live imaging of a control unarrested oocyte spindle; corresponds to Figure 2D (top row). Shown are GFP::tubulin and GFP::PLK-1 (green), and mCherry::histone (magenta). Oocytes were dissected into Meiosis Media containing vehicle and immediately filmed. The spindle maintains bipolarity in metaphase, shortens, rotates towards the cortex and then elongates to undergo anaphase. This phenotype was consistent in all videos (n=8). Scale bar = 5μm.

bioRxiv preprint doi: https://doi.org/10.1101/2024.08.03.606476; this version posted August 4, 2024. The copyright holder for this preprint (which was not certified by peer review) is the author/funder, who has granted bioRxiv a license to display the preprint in perpetuity. It is made available under aCC-BY 4.0 International license.

Video 4: Unarrested oocytes exhibit spindle defects following acute PLK-1 AID

Live imaging of an unarrested auxin treated oocyte spindle; corresponds to Figure 2D (bottom row). Shown are GFP::tubulin and GFP::PLK-1 (green), and mCherry::histone (magenta). Oocytes were dissected into auxin-containing Meiosis Media and immediately filmed. Upon auxin treatment the spindle elongates, chromosomes become misaligned and midspindle loses integrity. Spindle defects were observed in all videos (n=7). Scale bar = 5μm.

Video 5: Pre-treatment of oocytes with vehicle does not yield excess tubulin densities

Live imaging of a vehicle treated oocyte; corresponds to Figure 4A (top row). Shown are GFP::tubulin and GFP::PLK-1 (green), and mCherry::histone (magenta). Worms were soaked in vehicle-containing Meiosis Media for 30 minutes before oocytes were dissected and filmed. Control oocytes were able to complete meiosis I and successfully segregate chromosomes without spindle defects or excess tubulin density in the cell. This phenotype was consistent in all videos (n=7). Scale bar = 5μm.

Video 6: Pre-treatment of oocytes with auxin results in ectopic microtubule polymerization

Live imaging of an auxin treated oocyte; corresponds to Figure 4A (bottom row). Shown are GFP::tubulin and GFP::PLK-1 (green), and mCherry::histone (magenta). To achieve full PLK-1 depletion throughout the cell, worms were soaked in auxin-containing Meiosis Media for 30 minutes before oocytes were dissected and filmed. PLK-1 depleted oocytes formed tubulin-rich asters throughout the cell (n=7). Scale bar = 5μm.

Video 7: *klp-7(RNAi)* oocytes exhibit some microtubule asters when PLK-1 is present

Live imaging of tubulin density in a *klp-7(RNAi)* oocyte without auxin; corresponds to Figure 4B, middle row. Video is pseudocolored to show mean gray values of the GFP channel

bioRxiv preprint doi: https://doi.org/10.1101/2024.08.03.606476; this version posted August 4, 2024. The copyright holder for this preprint (which was not certified by peer review) is the author/funder, who has granted bioRxiv a license to display the preprint in perpetuity. It is made available under aCC-BY 4.0 International license.

(GFP::tubulin and GFP::PLK-1 signals). Oocytes were dissected into Meiosis Media containing vehicle and filmed. Control *klp-7(RNAi)* oocytes form some weak ectopic microtubule asters throughout the cell (n=8). Scale bar = 5µm.

Video 8: No significant tubulin asters are present throughout the oocyte immediately following acute PLK-1 AID

Live imaging of tubulin density in an auxin treated oocyte; corresponds to Figure 4B, top row. Pseudocolored video shows mean gray values of the GFP channel (GFP::tubulin and GFP::PLK-1 signals). Oocytes were dissected into Meiosis Media containing auxin and filmed. Microtubule asters were not observed immediately after acute AID since these oocytes were not pre-treated with auxin (n=7, same as Video 4 but zoomed out to view the entire oocyte). Scale bar = 5µm.

Video 9: *klp-7(RNAi)* oocytes form many ectopic microtubule asters following acute PLK-1 AID

Live imaging of tubulin density in a *klp-7(RNAi)* oocyte treated with auxin; corresponds to Figure 4B, bottom row. Pseudocolored video shows mean gray values of the GFP channel (GFP::tubulin and GFP::PLK-1 signals). Oocytes were dissected into auxin-containing Meiosis Media and filmed. Following auxin treatment, oocytes had increased tubulin density and multiple microtubule asters formed throughout the oocyte (n=8). Scale bar = 5µm.
